# The Recreation Rooms

**Published:** 1917-06-16

**Authors:** Ward Muir

**Affiliations:** R.A.M.C. (T.), 3rd London General Hospital.


### THE RECREATION ROOMS.

By LANCE-CORPORAL WARD MUIR, R.A.M.C. (T.), 3rd London General Hospital.

### Nigger Minstrels in a War Hospital.

WE rather pride ourselves at the 3rd London on the fame of our hospital, not merely as a place in which the wounded get well, but as a place in which they also "have a good time." The two things, truth to tell, are interlinked—a truism which might seem to need no labouring were it not for the evidence brought from more rigid and red-tape-ridden establishments. A couple of our most valued departments are the "Old Rec." and the "New Rec."—the old and new recreation rooms. The new recreation room, a spacious and well-built "hut," contains three billiard tables, a library, and current newspapers, British and Colonial. This room is the scene of whist-drives, billiard and pool tournaments, and other sociable ongoings. Sometimes there is an exhibition match on the best billiard table. The local champion of Wandsworth shows us his skill, and a very pretty touch he has. Once the lady billiard champion of England came, and defeated the best opponent we could enlist against her—an event which provoked tremendous applause from a packed congregation of boys in blue.

The old recreation room is fitted with a permanent stage for theatricals and concerts. It is also our "Movie Palace" (I think our hospital was the first to instal a cinematograph as a fixture). During the morning the floor area is dotted with miniature billiard tables, which are never for a moment out of use. In the afternoon these are removed, some hundreds of chairs replace them, and at 4.30 we begin an entertainment—music, a play (we have had Shakespeare here), lantern slides, films, or what not. Those entertainments, which have continued unbrokenly since the hospital began to function in 1914, constitute the outstanding feature of the "good time" enjoyed by 3rd Londoners. The "Old Rec." and its crowded concerts will be a memory cherished by hosts of fighting men from the homeland and from overseas.

### THE NEED FOR A DAY ROOM.

In the original hospital plan—drawn up before the war—the "Old Rec." (which is a part of the main school building) was marked down to be a ward of forty beds. Its structure, its internal geography, and the sheer impossibility of providing it with the essential sanitary conveniences would make it unsuitable to be a ward of four beds, let alone of forty. On this account its allotment for recreation purposes would be excusable. But the "Old Rec." and the "New Rec.," too, for that matter, justify their superficial waste of bed space on other, and unanswerable, grounds. It is a mere matter of common sense to arrange some centre to which the patient can repair and employ his leisure when he is sufficiently well to potter about, though not well enough to be discharged from hospital. Instead of idling in his ward and disturbing the patients who are still confined to bed, and who often are urgently in need of quietness, the convalescent departs to one or other of the recreation rooms; morning and afternoon, where he can make as much noise as he likes, and where he can meet and fraternise with his comrades from every Front. (What exchanging of stories those recreation rooms have witnessed!) On the one hand, then, the seriously ill patients are not annoyed by the rovings in the ward of the walking patients; and on the other the walking patients are not irked by the necessity for keeping quiet at a period when returning health stimulates them to a wholesome desire for fun. Both kinds of patients thus may legitimately be said to get better more quickly than they would have had a chance to do were it not for the recreation rooms. It is within the writer's knowledge that the medical staff of the hospital, on being consulted as to the "bed value" of the recreation rooms, unanimously agreed that their existence reduced the average sojourn of the hospital's inmates by a definite "per day" ratio: that ratio, so far from showing a bed-space waste, worked out at a per annum gain of bed space equivalent to a ward—if such a colossal ward could be conceived—of upwards of 300 beds. So much for a point which might not appear to be worth detailed explanation, but which has here been glanced at in order that critics (for, unbelievable though it sounds, there have been curmudgeons to growl of spoiling the wounded by too much pleasure) may be answered in advance. The recreation rooms are a paying investment, both to the hospital and to the State. This is our trump card in any "spoiling the wounded" controversy—though I dare say that most of us would not, in any case, care twopence whether the concerts and films and billiards were an investment or an extravagance—nothing would stand in the way of our ambition to provide the now proverbial "good time" for all the guests of the 3rd London.

### ENTERTAINMENTS INFINITE IN VARIETY.

Scores of concerts of an excellence which would have been noteworthy anywhere have been presented to our assemblages of wounded in the "Old Rec." Singers, musicians, actors, and actresses have come and given of their best. Miss Hullah's Music in War Time Committee (that delightful body), and Mr. Howard Williams's parties, are perhaps our greatest regular standbys. Certain sections of the public know Mr. Howard Williams's name as a famous one in other fields of activity: to thousands of soldiers it is honoured as that of the man who tirelessly organised scrumptious tea-parties, pierrot shows, exhibition boxing contests, nigger troupe entertainments—a list of jollifications, indoors in winter and in the open air in summer, infinite in variety, and guaranteed never once to fall flat. A curious Empire reputation this of Mr. Williams!

Recently, for instance, a nigger troupe visited the hospital. To be exact, they were the Metropolitan Police Minstrels ("by permission of Sir E. R. Henry, G.C.V.O., K.C.B., C.S.I., Commissioner"); but no member of the audience, I imagine, could picture those jocose blackamoors, with their tambourines and bones, as really being anything so serious as traffic-controlling constables. That their comic songs were accompanied by a faultless orchestra was understandable enough. One can believe in a police band. One is not surprised that the police band is a good band. To believe that the ebony-visaged person with the huge red indiarubber flexible mouth, who sings "Under the Archway, Archibald," and follows this amorous ditty with a clog dance, is—in his washed moments—the terror of burglars, requires unthinkable flights of imagination. As I gazed at this singular resurrection of Moore and Burgess and breathless childhood's afternoons at the St. James's Hall—the half-circle of inanely alert faces the colour of fresh-polished boots—the preposterous uniforms and expansive shirtfronts—the "nigger" dialect which this strange convention demands, but which cannot be said to resemble the speech of any African tribe yet discovered—I found that by no effort of faith or credulity could I pierce the disguise and perceive policemen.

A Reminiscent Entertainment.

It is at least twenty years since I met a nigger minstrel in the flesh. Vague ghosts of bygone persons and of piquant anachronisms seemed to float approvingly in the air; the Prince Consort, bustles, the high bicycle, sherry, Moody and Sankey, the Crystal Palace, Labouchere, "Pigs in Clover," Lottie Collins, Evolution, Bimetallism—hosts of forgotten images, names, and shibboleths came popping out from the brain's dusty pigeon-holes, magically released by the spectacle of the nigger troupe.

Yes, I was indeed switched into the past by Mr. Bones, Massa Jawns'n, and the rest. And yet the present might have seemed more emphatic and more poignant. One felt rather than saw an audience of several hundred persons in the dim rows of chairs. And laughing at the broad witticisms of the niggers, or enjoying their choruses and orchestral accompaniments, one forgot just what that half-glimpsed audience consisted of, what it meant, and how it came to be here assembled.

Of course, when the lights were turned up in the interval, one beheld the usual spectacle—stretchers, wheeled chairs, crutches, bandaged heads, arms in splints, blind men, men with one arm, men with one leg; rank on rank of war's flotsam and jetsam, British, Australians, New Zealanders, Newfoundlanders, Canadians, come to make merry over the minstrels; in the front row the Colonel and the matron, with officer patients; here and there an orderly or a V.A.D.; here and there a sister with her "boys." It was a family gathering. I descried no strangers, and no one not in uniform, unless you count the men too ill to don their blue slops; these had been brought in dressing-gowns or wrapped in blankets. No mere haphazard audience, this, of anybody and everybody who chooses to pay at a turnstile! Entrance to this hall is free . . . but the price is beyond money, all the same.

A family party it was, decidedly. Thick fumes of tobacco smoke uprose from it. (Shall we ever abandon the cigarette habit now?) Orderlies continued to arrive and stow themselves discreetly in corners; by some strange providence each orderly had found that for a while he could be spared from ward or office. Staff-sergeants, sergeants, corporals—mysteriously they made time to leave their various departments. Even a bevy of masseuses (those experts eternally on the rush from ward to ward) had peeped in to see the nigger minstrels. And everybody was pleased; every jest and every conundrum got its laugh, every ballad its applause. Not that we ever "give the bird" to those who come to amuse us. Offer us skill in any shape or form—pierrots, niggers, pianist, violinist, conjurer, ventriloquist, dancer, reciter; any or all of these will be appreciated warmly.

For the nigger minstrels there were no empty chairs until, in the midst of Part II. ("A Laughable Sketch"—*vide* the programme—wherein female *rôles* were doubly coy by reason of the masculinity of their falsetto dialogue and remarkable ankles), a messenger stole hither and thither, whispering to the orderlies, who promptly tiptoed from the room.

A convoy of new arrivals demanded our presence. The silent ambulances were gliding up to the entrance of the hospital. Orderlies, fetched from their jobs and from the entertainment, lined up in the rain to take their places in the quartettes of bearers who lifted out the stretchers. The assistant matron, standing in the shelter of the door, checked her list; the medical officer handed out the ward tickets; the lady clerks from the admission and discharge office took the patients' particulars. And the bathroom became very busy.

As I started to wheel a much-bandaged warrior to his ward, the recreation-room door opened and a burst of music-cum-essence-of-nigger emerged on his astonished ears. I was a little doubtful as to whether our new guest would not think his reception somewhat flippant in key. The poor fellow was visibly suffering, and the sound of tambourines and comedians' guffaws seemed a scarcely proper comment on his condition. I might have spared myself these misgivings. "Say, chum," he interrogated me feebly, "what's that noise?" "Nigger minstrels, old man." "Golly! and have I got to go straight to my bed?"

Alas! he had to. It would be long before he could be well enough to be taken to one of our entertainments. But, had he been given his way, he would have gone direct from his fatiguing overseas journey into the Old Rec. to join the family party and chuckle at Mr. Bones and Massa Jawns'n. . . . No doubts assailed *his* mind as to whether it was right to "waste bed-space" on mere frivolities. A nigger-minstrel show was to him a deal more important, in fact, than his wound. And perhaps, in instinct, he was not far wrong.

